# On Resection of the Tarsal and Metatarsal Bones in Caries of the Foot,—as a Means of Avoiding Amputation

**Published:** 1855-03

**Authors:** Henry H. Smith

**Affiliations:** Consulting Surgeon to the Philadelphia Hospital


*On Resection of the Tarsal and Metatarsal Bones in Caries of the Foot,—as a means of avoiding amputation. Illustrated by a case of resection of two of the cuneiform and two of the metatarsal bones—cured.* By HENRY H. SMITH, M.D., Consulting Surgeon to the Philadelphia Hospital.

With the exception of the vertebræ, few of the short or thick bones are more liable to caries or to tuberculous deposit than those of the foot. When developed in these bones, caries seldom extends itself rapidly, but rather excites such inflammatory action in the surrounding parts, as tends to circumscribe and limit the disorder to the bone in which it originated. Although often accompanied by considerable constitutional disturbance, as well as by swelling and inflammation of the soft tissues, the ultimate tendency of caries of the bones of the foot is towards a cure; so that when the absorbents are aided in the removal of the diseased bone, the soft parts often again resume their natural appearance and perform their normal function. Caries of the tarsal bones may therefore be regarded as a disorder of a partial character, as it does not involve an extended surface. According to Mr. Miller,* all carious bones present three parts: "1st, a central ulcerous cavity. 2d, a circle exterior to this, which is affected by interstitial absorption; and 3d, a portion (which is of comparatively sound bone,) in which there is a low grade of action of the sthenic character. The two interior circles are, he remarks, "incapable of efficient reproduction or repair; but the external ones atone for their deficiency by throwing out new osseous matter."

If, then, in caries of the foot, the elimination of the diseased structure be aided by incising the soft parts, perforating the exterior shell of bone, and scooping out or otherwise removing the diseased particles, healthy granulations will arise from the outer circle of the bone, and the patient's general health be improved by the diminution of the otherwise long period of confinement.

But in addition to the time saved by this means of treating caries of the bones of the foot, the patient will also be materially

* Miller's Principles of Surgery, American Edition, p. 44.

benefitted by the preservation of that portion of his lower extremity which is essential to active or graceful progression. In amputation at the metatarso-tarsal articulation, either as practised by Hey or by Lisfranc, the patient is necessarily deprived of the length of a portion of the limb which is intended by nature to aid him in progression in the erect position; whereas by resection this is preserved, and a steady support in walking guaranteed, as will be apparent from a slight reference to the structure of the foot. If the function of the foot was merely to aid in the support of the body in the erect posture, its length would probably not have been extended beyond the tarsus, as the arched form of these bones, together with their close apposition, indicate that they alone would capable of furnishing a vertical support. But the greater length of the metatarsal bones, as well as those of the phalanges when unrestrained by a boot, as clearly demonstrate that their function must be different, and show that they are mainly intended to aid progression, their length and position in advance of the tarsus exhibiting not only the creation of an abutment for the anterior support of the tarsal arch, but also the formation of such a series of moveable props as must add materially to the preservation of the proper centre of gravity in walking. In the Chinese women, where the development of these anterior props is prevented by constriction, the gait becomes unsteady and toddling, whilst in cases of amputation of both feet, it has generally been found necessary to compensate for their loss by the introduction of cork or some other material into the front of the boot. The bones of the tarsus have on the contrary been frequently removed without materially impairing progression, numerous instances of the resection of the astragalus, cuboid and scaphoid, having been reported in which there was little destruction of stability in any forward movement in the erect posture.

As evidence of the value of resection in the preservation of this important anterior portion of the foot, the following cases are now presented.

*Caries of the Cuneiforme Internum and Medium, as well as the first two Metatarsal Bones, consequent on injury—cured with a useful foot, by resection.*

Francois C., a Frenchman, æt. 39 years, was engaged in May last as a wood-cutter. In June 1854, after having twice bruised his instep, he was attacked with inflammation of his left foot, attended by great swelling and stiffness. Notwithstanding the pain, he continued working until the effusion gathered and broke directly over the os cuneiforme internum. Being compelled to give up his work, he entered the Philadelphia Hospital early in Oct., 1854, and was treated for his injury, but without much amelioration of his symptoms. In the latter part of this month I saw him and obtained the preceding account. The foot at this time was very much swollen from the ankle to the toes; was of a deep purple color, interspersed with spots of a brighter red, and had upon its dorsum one fistulous orifice over the os cuneiforme internum, another over the upper third of the first metatarsal bone, a third over the cuneiforme medium, and a fourth between the second and third metatarsal bones, or rather in the second interosseous space. A probe introduced into each of these openings readily entered the structure of the bones. There was no motion in the part, and there was a free, sanious discharge, with marked inflammatory disorder of the soft tissues. The constitutional symptoms were loss of appetite, deranged digestion, and quick pulse, (due to pain, &c.,) and his general habits were only moderately temperate. As I thought that the circumstances seemed to render it advisable to operate and remove the diseased bone, and he declined submitting to amputation till all other means had failed, I decided to resect the first metatarsal bone entire and scrape out the greater portion of the cuneiforme internum, hoping that the removal of these portions would eradicate most of the disease. This I accordingly accomplished in the usual manner, before the medical class, on November 15th. Under the use of the tepid water dressing, the wound did well, and the parts healed kindly, much of the swelling disappearing, but as the other fistulous orifices continued open and the bones beneath them were also diseased, that is, the cuneiforme medium and second metatarsal, I decided to resect them also, which I did on Dec. 8th, by a careful incision on the dorsum of the foot, the tendon and anterior tibial artery being pushed to one side. After scooping out the cuneiforme medium I removed the head and upper third of the second metatarsal bone, and dressed the wound as before. Little or no trouble supervened; the wound healed kindly; the inflammatory action diminished, and on

January 12th, Francois was walking about the ward, improved in health and with every appearance of a useful foot. The depression left by the removal of the bones has been much diminished by deposition and lateral approximation, and the toes are now comparatively useful, much to the patient's gratification, especially as "he has escaped amputation."

A somewhat similar operation was performed by Dr. Geddings, of Charleston, and reported by him in the North American Archives of Medical and Surgical Science, vol. 1, p. 36, Baltimore, 1835. As this case is briefly stated, and is not perhaps readily accessible to the reader, I shall quote it somewhat at length:

"Case of Necrosis of the first three metatarsal and the cuneiform bones—extirpation of them, leaving the corresponding toes; cure."—A boy aged 18 years had his foot enormously enlarged, with several fistulous openings on the instep and inner border of the foot, through which a considerable quantity of ill-conditioned matter was discharged. The probe indicating that the bones were diseased, Dr. Geddings operated as follows:

An incision being made along the inner side of the foot from one extremity of the first metatarsal bone to the other, and its attachments having been destroyed by disease, the bone was readily removed so as to leave the great toe and its proper flexor and extensor tendons entire, and though the cuneiform bones as well as the second metatarsal bone were now found to be diseased, it was thought they might be thrown off by nature. The wound was therefore filled with dry lint, and the foot properly dressed.

As new fistulous orifices formed, and nature did not accomplish the removal, Dr. Geddings decided on a second operation, which he performed as follows: An incision being made in a longitudinal direction, upon the dorsum of the foot and in the course of each metatarsal bone, the lips were separated so as to expose the extensor tendons, and these being drawn aside, the bones were disarticulated first in front and then behind, and dissected from their beds so as to leave the toes with their tendons. This done, the three cuneiform bones were removed, as they could be easily picked away in fragments, in consequence of the progress of the disease. After a few days, suppuration was established, healthy granulations sprang up, the tumefaction of the foot subsided, and the preservation of the toes was found highly advantageous to the patient in walking, which he was able to do with but a trifling impediment in his step.*

In reporting this case, Dr. Geddings mentions, that it had been pronounced to be one requiring amputation of the leg, "an operation which it is to be feared is often resorted to on account of diseases of the tarsal and metatarsal bones, which might in many instances be relieved by a procedure which would secure to the patient the use of his foot."

Although twenty years has elapsed since the publication of this case by Dr. Geddings, the one which I have reported in this paper is, I believe, the first in which the means of avoiding amputation of the foot proposed by him, has been repeated, or, at all events, published. As a plan of affording relief without creating deformity, the resection of these bones certainly deserves more general consideration among American surgeons than it has apparently received. That the tumefaction and other inflammatory affections of the soft parts which accompanies the disease of these bones, has created anxiety in the minds of some surgeons as to the extent of the disorder, is evident, as nothing else could have induced the performance of amputation. Further investigation on the part of those who have hitherto looked favorably on amputation as a means of relieving a patien under these circumstances, will, however, show, that most of these appearances of great disorder are only skin deep; that the affection of the bones which originate it, is circumscribed, and that by the removal of the cause, the remainder of the member may be preserved. In the form of caries usually described as "scrofulous disease" of the bones of the foot, there is often only the deposit of a solitary tubercle in the cancellated structure, and it has been well shown by Nelaton†, that the progress of tuberculous matter when deposited in bone is similar to that noticed when it is deposited in the lungs; that is, to irritate and inflame the surrounding parts, and thus lead to the suppuration and disintegration of tissue, until it is thrown off. But in the disin-

* Dr. Geddings, loc. citat.

† Recherches sur l'Affection Tuberculeuse des os. Paris, 1837.

tegrating process as seen in bone there is a marked tendency in the pus to discharge towards the surface instead of invading the neighboring articulations, and hence resection of the bones of the foot presents us, even in these cases, with a means of eradicating the diseased structure, and yet preserving sufficient of the adjacent sound parts to enable the patient to use the foot.

